# Food consumption pattern and dietary diversity of a vegetarian population in Ghana

**DOI:** 10.4314/gmj.v55i1.5

**Published:** 2021-03

**Authors:** Matilda Asante, Benjamin Frimpong, Freda Intiful, Portia Nkumsah-Riverson, Somah A Nkansah, Boadiwaa Ofori-Amanfo, Yaunuick Y Dogbe, George A Asare

**Affiliations:** 1 Department of Dietetics, School of Biomedical and Allied Health Sciences, University of Ghana, Accra, Ghana; 2 Department of Medicine, Adventist Hospital, Kumasi, Ghana; 3 Department of Dietetics, 37 Military Hospital, Accra, Ghana; 4 Department of Medical Laboratory Sciences, School of Biomedical and Allied Health Sciences, University of Ghana, Accra

**Keywords:** Food consumption pattern, dietary diversity, vegetarians

## Abstract

**Objective:**

This study examined the food consumption pattern and dietary diversity of a vegetarian population in the Greater Accra Region of Ghana.

**Methods:**

A cross-sectional study was employed to examine the nutritional status of four (4) vegetarian groups in the Greater Accra Region of Ghana. One hundred and twenty-two (122) vegetarians were recruited using the total enumeration technique. Food consumption pattern and dietary diversity were assessed using a validated qualitative food frequency questionnaire and a 24-hour dietary recall, respectively. Dietary diversity was calculated using the FAO guidelines.

**Results:**

Sixty eight percent (68%) of the vegetarians reported daily intakes of vegetable protein. Majority of the vegetarians (80.6%) reported daily intakes of cereals and grains while 54% reported daily intakes of tubers. Eighty two percent (82%) and 72% of the vegetarians consumed vegetables and fruits on daily basis respectively. A few of the vegetarians (29%) reported daily intakes of fruit juices. Soft drinks, deep fried foods and fast foods were occasionally consumed. About 40.3% of the vegetarians obtained a dietary diversity score of four (4). Majority of them (68.9%) had low dietary diversity.

**Conclusion:**

The vegetarians had low dietary diversity which may lead to inadequate nutrient intakes. Thus, there is the need for nutrition-related professionals to give appropriate information on a vegetarian diet and educate vegetarians to include a variety of foods in their diet.

**Funding:**

None declared

## Introduction

Vegetarian diets have become increasingly popular in recent times.^1^ A vegetarian is someone who consumes a diet consisting mostly of plant-based foods, including fruits, vegetables, legumes, nuts, seeds, and grains with or without eggs, dairy products or both.^2^ There are an estimated 1.5 billion vegetarians worldwide, either by choice or of necessity.^3^ Craig reported that the market for processed vegetarian food has grown significantly during the past decades in the United States .^4^ The 2008–2012 National survey and 2016 Ipsos Mori survey in the United Kingdom also showed that 1.7 million of the country's population were vegetarians and vegans.^5^,^6^ In addition to the growing number of vegetarians, increasingly more people in Western countries continue to reduce their meat consumption.^7^

Vegetarian dietary patterns have been defined based on the absence of certain animal foods in the diet^8^; however, they differ greatly with respect to the consumption of many other food groups. Vegetarianism encompasses a spectrum of eating patterns: from diets that exclude all animal meats and products (vegans), and to diets that include eggs (ovo-vegetarians), milk and milk products (lacto-vegetarians), fish and fish products (pesco-vegetarians) or a combination.^9^ Within each pattern, there might be considerable variation regarding the extent to which animal products are excluded.^10^ Dairy and eggs are the most consumed animal protein products and are considered acceptable in many vegetarian dietary patterns.^11^

People select vegetarian diet for several reasons including the desire for better health, ethical concerns, environmental consideration and religious belief. These underlying motivations may influence the choice of food consumed, beyond the avoidance of meats and other animal foods.^8^ Dietary patterns of vegetarians have been associated with several favourable health outcomes in epidemiological studies, including the Adventist Health Study 2 (AHS-2).^4^ A position statement by the Academy of Nutrition and Dietetics reports, “appropriately planned vegetarian, including vegan, diets are healthful, nutritionally adequate, and may provide health benefits for the prevention and treatment of certain diseases.^12^ Despite the numerous health benefits of vegetarian diets, it is also established that vegetarianism is generally associated with some nutritional deficiencies, especially when the elimination includes many foods, and not only animal foods. An inappropriately planned vegetarian diet may be low in nutrients such as proteins, calcium, iron, zinc, vitamin B^12^ and omega-3 fatty acids.^4^, ^13^ Adequacy of a vegetarian diet can therefore be ensured through the intake of a diversified diet. According to Orlich et al, differences in food consumption dietary pattern and diversity may be important in determining the implications of vegetarian diets on health.^8^

Currently, there is no national data on the number of vegetarians in Ghana. There is, however, an indication that vegetarianism is gradually increasing, and this is evidenced by increase in vegetarian restaurants and the establishment of Vegetarian Associations in the Greater Accra region of Ghana. The Ministry of Health (MOH) in partnership with a non-governmental organization (Africa Hebrew Development Agency, (AHDA)) in recent years initiated the Regenerative Health and Nutrition Programme (RHNP) that emphasized among other things the consumption of plant-based diets in Ghanaian households. The RHNP is modeled on the practices of the Africa Hebrew Israelites of Jerusalem who are reported to have low risk of non-communicable diseases.^14^ Data on food consumption patterns of Ghanaian vegetarians is limited. We examined food consumption pattern and diversity of a vegetarian population in Ghana.

## Methods

### Study Design, Setting and Population

This study was a cross sectional study that examined the nutritional status of four vegetarian groups in the Greater Accra Region of Ghana. The study sites were Weija, Mamprobi, Madina and Pokuase found in four (4) municipal districts in the Greater Accra Region of Ghana (Ga South Municipal, Accra Metropolitan, La Nkwantanang Municipal and Ga West Municipal respectively). For each study site, the total enumeration technique was used to recruit participants.

Individuals who reported that they were vegetarians, aged 18–75 years participated in the study. Individuals who had practiced vegetarianism for more than one year were included while those who refused to participate were excluded.

### Data Collection

Socio-demographic information and usual food intakes of the participants were obtained using a structured questionnaire. Anthropometric measurements were also taken.

### Sociodemographic information

Information included age, marital status, educational level, employment status and lifestyle behaviour of participants as well as number of years they had practiced vegetarianism, and type of vegetarianism (vegan, lacto, ovo, ovo-lacto, pesco etc).

### Anthropometric Measurement

#### Height

Height was measured with a Seca stadiometer to the nearest 0.1cm. Patients stood on the level platform of the stadiometer with barefoot, with their knees straightened. The lower eye socket to the ear called the Frankfurt Plane, was ensured to be perpendicular to the vertical board of the stadiometer. The sliding piece of the stadiometer was moved gently to the level of the crown of their head, before readings were taken.

#### Weight and Body Composition Assessment

Weight and body composition was measured using an Omron body composition monitor (HBF-516B model) following standardized procedures. The equipment was turned on and patient's height and gender imputed. Patients stood barefooted with straightened knees and back on the platform of the monitor looking straight ahead. It was ensured that each foot was placed on the foot and heel electrodes. Percentage body fat, muscle mass, visceral fat, body mass index (BMI) and total body water were recorded.

### Dietary Assessment

#### 24- Hour Recall

The 24-hour dietary recall was used to capture detailed information about all foods and beverages consumed by the participants in the past 24 hours. Timing of meals, source of food and portion size of each food and beverage were captured. Household measures were used in estimating the portion sizes.

### Food Frequency Questionnaire

A validated qualitative food frequency questionnaire (FFQ) adopted from Owusu et al^15^, was used to assess the food consumption pattern/ usual food intakes of participants over a month. The FFQ was originally designed to assess commonly consumed foods by the Ghanaian population. The questionnaire was revised to include foods commonly consumed by vegetarians. It consisted of a food list with estimates of frequency of consumption from seven food groups. This comprised of animal protein; plant protein; cereals and grains; tubers; vegetables, fruits and fruit juices; soft drinks and fast foods, deep fried foods and pastries. Participants were asked to indicate the frequency with which each food was consumed (daily, 1–2x a week, occasionally and never). The questionnaire was pretested in a similar population and found to be feasible to use in the main study. Participants in the pretest were not included in the main study.

### Dietary Diversity Score

Dietary diversity score was calculated using data obtained from a one day 24-hour dietary recall. Foods consumed were grouped into nine, starchy staples, dark green leafy vegetables, other vitamin A rich fruits and vegetables, other fruits and vegetables, organ meat, meat and fish, eggs, legumes, nuts and seeds and milk. Zero (0) was awarded to participants who did not consume food from a food group while one (1) was awarded to consumption from a food group. The total scores for dietary diversity were obtained by adding up from the individual food groups. The maximum score that would be obtained was nine (9). Dietary diversity was calculated using a simple count of food groups consumed over the period using Food and Agriculture Guidelines^15^. Scores of five (5) and above were categorized as adequate nutrient intake (high dietary diversity score) scores below five (5) were categorized as low nutrient intakes (low dietary diversity score). Scores were based on the mean DDS obtained.^16^

### Data Handling

Data was treated with a high level of confidentiality. In order to ensure anonymity, identifiers and special codes were used. Data obtained from the study was stored on password-protected computer and made available only to research team.

### Data analysis

Data was analyzed using the Statistical Package for Social Sciences (SPSS version 20.0) and reported with descriptive statistics such as means, standard deviations, frequencies and percentages. Chi-square statistics was used to examine the relationship between explanatory variables and outcome variable. Fischer's exact test for observations less than five. All statistical tests were considered significant at p< 0.05.

### Ethical consideration

This study was approved by the Ethics and Protocol Review Committee of the School of Biomedical and Allied Health Sciences, University of Ghana (SBAHS/10414067/AA/ND/2015-2016). Informed consent was obtained from each participant before enrollment into the study.

## Results

### Socio-demographic characteristics of participants

Table 1 shows the socio-demographic characteristics of the 122 participants in the study. The mean age was 48.27 ± 15.4 years with the male vegetarians being significantly older than the females (p = 0.009). More than half of the vegetarians were married and employed. Majority of the vegetarians (89.5%) were Christians. Fifty percent (50%) of the participants had attained tertiary level education with males constituting a significantly higher proportion (p=0.049). Smoking and alcohol intake were not common among the vegetarians.

**Table 1:** Socio-demographic characteristics of participants

	Male (n=86)	Female (n=36)	Total (n=122)	
Variable	n (%)	n (%)	n (%)	*P*-value
**Age in years** **(Mean ±SD)**	50.63 ±15.13	42.64 ±14.77	48.27±15.40	0.009*
**Marital status**				
**Single**	19 (22.1)	12 (33.3)	31 (25.4)	0.174
**Married**	58 (67.4)	17 (47.2)	75 (61.5)	
**Divorced**	5 (5.8)	2 (5.6)	7 (5.7)	
**Widowed**	4 (4.7)	5 (13.9)	9 (7.4)	
**Education**				
**No formal education**	5 (5.8)	3 (8.3)	8 (6.6)	0.049*
**Basic education**	14 (16.3)	14 (38.9)	28 (23.0)	
**SHS/‘O’ level**	20 (23.3)	5 (13.9)	25 (20.5)	
**Tertiary**	47 (54.7)	14 (38.9)	61(51.0)	
**Religion**				
**Christian**	77 (89.5)	34 (94.4)	111 (91.0)	0.001*
**Muslim/Hindu**	9 (10.5)	2 (5.6)	11 (9.0)	
**Employment**				
**Employed**	48 (55.8)	21(58.3)	69 (56.6)	0.425
**Unemployed**	9 (10.5)	2 (5.6)	11 (9)	
**Retired**	22 (25.6)	7 (19.4)	29 (23.8)	
**Student**	7 (8.1)	6 (16.7)	3 (10.7)	
**Lifestyle Behaviour**				
**Smoking**	1 (1.2)	0 (0)	1 (0.8)	0.402
**Alcohol intake**	3 (3.5)	1 (2.8)	4 (3.3)	0.288

### Years and Type of Vegetarianism

**Figure 1:**
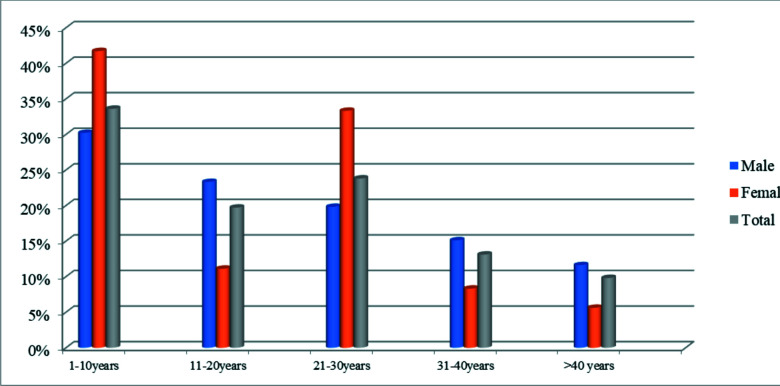
Years of vegetarianism

Figure 1 shows that about one-third (33.6%) of the vegetarians had practiced vegetarianism for ten (10) years or less.

**Figure 2:**
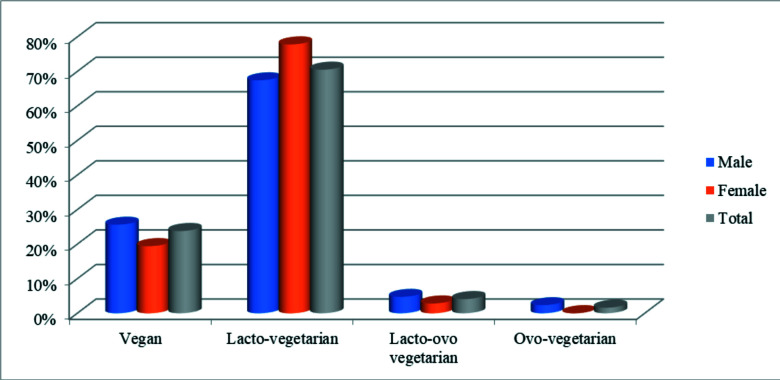
Types of vegetarians

Furthermore, from 30 years and above, majority of them were males. Most of the participants (78.0%) reported that they were lacto-vegetarians (Figure 2).

### Reasons for Vegetarianism

**Figure 3:**
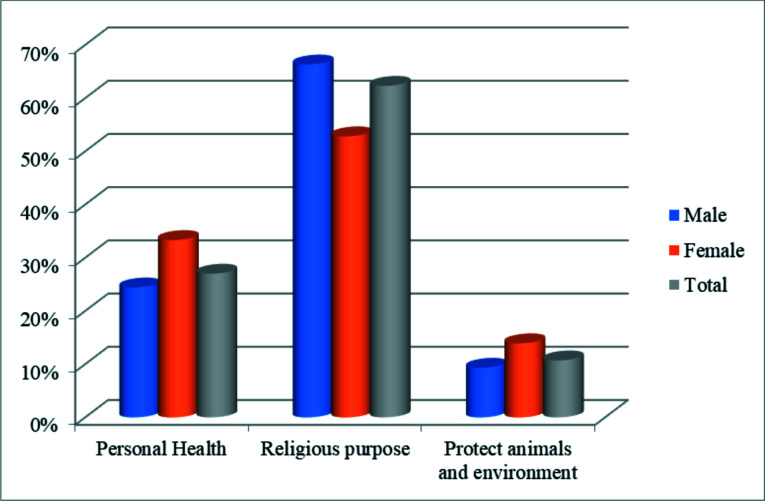
Reasons for vegetarianism

Approximately 62% of the participants reported that their religious belief and practices made them follow vegetarianism. Thirty-three percent (33.0%) reported adoption of vegetarianism for health reasons (Figure 3).

### Body Composition of the Participants

The mean BMI (25.16 ± 4.26) kg/m^2^ of the participants was slightly above the normal range. There were significant differences in BMI (p = 0.001), body fat percentage (p=0.001) and height (p=0.001) between the genders (Table 2).

**Table 2:** Body composition of the participants

	Male (n=86)	Female (n=36)	Total (n=122)	
Variables	Mean (SD)	Mean (SD)	Mean (SD)	*P*-value
Height	1.70 (0.06)	1.61(0.06)	1.67(0.07)	0.001*
Weight	69.82(11.74)	71.29 (13.52)	70.25 (12.26)	0.573
BMI	24.28 (3.83)	27.28 (4.50)	25.16 (4.26)	0.001*
Visceral fat	8.28 (4.75)	8.89 (3.30)	8.47(4.37)	0.424
Body fat	20.30 (8.35)	37.28 (9.38)	25.32 (11.61)	0.001*

### Frequency of consumption of foods among participants

One hundred out of the 122 participants fully completed the food frequency questionnaire. Sixty eight percent (68%) of the vegetarians reported daily intakes of vegetable protein (cowpea, beans, groundnut and cashew nut). Concerning animal protein intake, 26.0% of the vegetarians consumed milk daily. Eggs and seafood were never consumed (Table 3). The vegetarians, however, consumed comparatively more cereals and grains (73.0%) than tubers (54.0%).

**Table 3:** Frequency of consumption of food among participants (N=100)

Foods	Daily	1–2 times a week	Occasionally	Never
	n (%)	n (%)	n (%)	n (%)
**Animal protein** a	26 (26.0)	28 (28.0)	23 (23.0)	100 (100.0)
**Vegetable protein** b	68 (68.0)	28 (28.0)	4 (4.0)	0 (0)
**Cereals and grains**	73 (73.0)	26 (26.0)	1 (1.0)	0 (0)
**Tubers**	54 (54.0)	39 (39.0)	6 (6.0)	1 (1.0)
**Vegetables**	82 (82.0)	15 (15.0)	3 (3.0)	0 (0)
**Fruits**	72 (72.0)	24 (24.0)	4 (4.0)	0 (0)
**Fruit juices**	29 (29.0)	13 (13.0)	38 (38.0)	20 (20.0)
**Soft drinks**	9 (9.0)	21 (21.0)	49 (49.0)	21 (21.0)
**Fast foods**	0 (0)	6 (6.0)	14 (14.0)	80 (80.0)
**Deep fried foods**	12 (12.0)	30 (30.0)	30 (30.0)	28 (28.0)
**Pastries**	6 (6.0)	21 (21.0)	39 (39.0)	34 (34.0)

a Milk

b Cowpea, soyabeans, nuts

Majority of the participants consumed vegetables (82.0%) and fruits (72.0%) daily. Only 29.0% and 9.0% of the vegetarians consumed fruit juice and soft drinks daily respectively (Table 3). About 12.0% and 6.0% of the vegetarians reported daily consumption of deep-fried foods and pastries respectively. Majority of the participants (80.0%) reported never consuming fast foods (Table 3).

### Dietary Diversity

Table 4 shows the percentage of participants who consumed foods from each food group. Dietary data from 77 participants out of the 122 vegetarians who completed the 24-hour dietary recall were used to determine dietary diversity. All the participants (100%) consumed starchy staples. A higher proportion of the vegetarians (84.4%) consumed other fruits and vegetables such as orange, banana and pawpaw over the period. The least consumed food group was meat, fish, and organ meat.

**Table 4:** Percentage of participants who consumed foods from each food group

Food group	Male (n=60)	Female (n=17)	Total (n=77)
	n (%)	n (%)	n (%)
Starchy staple	60 (100.0)	17 (100.0)	77 (100.0)
Dark green vegetables	30 (50.0)	9 (52.9)	39 (50.6)
Other Vitamin A fruits and vegetables	30 (50.0)	6 (35.3)	36 (47.8)
Other fruits and vegetables	52 (86.7)	13 (76.5)	65 (84.4)
Organ meat	0 (0)	0 (0)	0 (0)
Meat and fish	6 (10.0)	2 (11.8)	8 (10.4)
Egg	1 (1.7)	0 (0)	1 (1.3)
Legumes, nut and seeds	40 (66.7)	10 (58.8)	50 (64.9)
Milk and milk products	24 (40.0)	11 (64.7)	35 (45.5)

### Dietary Diversity Score of Participants

About 40.0% of the vegetarians obtained a dietary diversity score of 4, while 7.8% of them obtained the highest dietary diversity score of 6 (Table 5). The mean dietary diversity score of the participants was 4.04 ± 1.02. Majority of them had low dietary diversity (68.9%).

**Table 5:** Dietary diversity score of the participants

	Male (n=60)	Female (n=17)	Total (n=77)
Diversity score	n (%)	n (%)	n (%)
**1**	0 (0)	0 (0)	0 (0)
**2**	4 (6.7)	1 (5.9)	5 (6.5)
**3**	14 (23.3)	3 (17.6)	17 (22.1)
**4**	22 (36.7)	9 (52.9)	31(40.3)
**5**	15 (25)	3 (17.9)	18 (23.4)
**6**	5 (8.3)	1 (5.9)	6 (7.8)
**7**	0 (0)	0 (0)	0 (0)
**8**	0 (0)	0 (0)	0 (0)
**9**	0 (0)	0 (0)	0 (0)
**Mean dietary diversity** **score**	4.05 ±1.05	4.00 ± 0.94	4.04 ±1.02

## Discussion

Current evidence shows that vegetarianism is increasing globally for religious, health and environmental reasons.^15^ It is maintained that a well-planned vegetarian diet is linked with healthy nutritional status. Again, vegetarian lifestyle and dietary practices are associated with prevention of diseases such as ischemic heart disease, type 2 diabetes and obesity.^17^,^18^,^19^ Some researchers have also reported micronutrient deficiencies with vegetarian dietary patterns.^4^, ^13^

Although vegetarian dietary patterns have been defined based on the absence of animal foods in the diet, they differ greatly with respect to the consumption of many other food groups. Several studies have been conducted on vegetarianism in Western countries. However, similar studies conducted in Ghana remain few. The aim of this study therefore was to examine the food consumption pattern of a vegetarian population in the Greater Accra Region of Ghana and determine the diversity of their dietary intakes.

In this present study, higher proportions of participants were males and older than their female counterparts. This is contrary to findings from other studies, which found that females tend to adopt vegan and vegetarian diets than males because they were more conscious of their weight and body shape.^20^ This finding is also not consistent with a finding among self-identified vegetarians in the United States where more females were found to be vegetarians than the males.^11^

With regards to religion, most of the participants were Christians. This was expected since many of the participants recruited were Seventh Day Adventists (data not shown). This denomination encourages its members to adopt a vegetarian lifestyle. Majority of the participants were lacto-vegetarians. This finding is not in accordance with another study conducted in US, which found out that most Adventist vegetarians were lacto-ovo.^21^ Most of the vegetarians were educated to the tertiary level with a significantly higher proportion of males than females having tertiary education. A study among vegetarians in the USA also reported similar findings.^20^ These findings suggest that more educated and knowledgeable individuals are more conscious of their health and have positive attitudes towards good nutrition. It may also be associated with their religious affiliations. Couceiro et al, stated that the motivations that led most religious institutions to promote vegetarian diets are based on health issues.^10^

Some studies found health as the main reason for choosing vegetarianism.^22^,^23^ Similarly, this present study showed that religious belief and practices were the main reason for practicing vegetarianism. However, this finding is not consistent with findings from a study conducted among vegetarians in the United States of America, Canada, and United Kingdom, which reported that the main motivator for participant's vegetarianism was health and well-being.^23^

Lifestyle behaviours such as smoking and alcohol consumption are very important factors to be considered in our health. Smoking and alcohol consumption were not common among the participants.

Low rates of smoking and alcohol consumption was also observed among German vegans.^24^ Study has shown that healthy lifestyle factors, particularly abstinence from smoking and alcohol intake, are important determinants of reduced mortality among those who follow a healthy lifestyle.^25^

Previous reviews have reported healthy body weight in vegetarian populations.^26^,^27^, ^28^ Similar observations were found in this study. Randomized control trials suggest that dietary factors influencing energy intake and possibly thermic effect of food may be responsible for these differences. Owing to the evidence that vegetarians have normal body weight and lower rates of obesity, and lower risk of non-communicable diseases.^29^

The major sources of protein among the vegetarians were vegetable proteins such as soya bean, cowpea, a*gushi*e (melon seeds) and nuts. Similar findings were found among vegetarians in Australia.^2^ The authors reported that the vegetarians consumed high amounts of nuts, legumes and seeds, which are good sources of protein. Few vegetarians in this study consumed animal protein. Some either consumed milk daily or once or twice a week. Eggs and seafood were never consumed. This finding is therefore not in agreement with findings from a study conducted in the USA which reported that majority of the vegetarians consumed some type of animal protein highlighting dairy and eggs as the most consumed animal products.^11^ In this present study, milk was the main source of animal protein. This was expected since more than half of the participants reported that they were lactovegetarians.

Some studies have reported a higher intake of cereals and grains among vegetarians^8^,^11^ These results were similar to findings from this present study. Cereals and grains were consumed more frequently than tubers. The vegetarian food pyramid adopted from Loma Linda University recommends a higher intake of cereals and grains. Higher intakes of cereal fibre and whole grain diets are associated with a reduced risk of type 2 diabetes, obesity and heart diseases.^29^,^30^

The higher intake of fruits and vegetables in this study is consistent with findings from previous studies.^31^,^32^ This practice is desirable since diets high in fruits and vegetables are rich sources of fibre, potassium, magnesium and other minerals and have the tendency to reduce body weight, blood viscosity and lower blood pressure.^33^ Additionally, vegetables are more nutrient-dense and lower in calories which is helpful for weight management.

Fast foods and deep-fried foods such as fried yam and plantains were not commonly consumed by the vegetarians. Fast foods and deep-fried foods are very high in trans-fatty acids. High intake of trans-fatty acids has been reported to decrease high-density lipoprotein and raise low-density lipoprotein cholesterol levels.^34^ This result shows the health consciousness of the vegetarians.

A poorly planned vegetarian diet that is filled with refined carbohydrates and fats may contribute excess calories and create nutrient deficiencies.

Majority of the vegetarians (68.9%) consumed food items from ≤ 4 food groups thus indicating low dietary diversity according to WHO/FAO guidelines.^16^ A smaller proportion of the participants (7.8%) consumed a diversified diet from six (6) food groups. Increase in an individual's dietary diversity score is associated with increased nutrient adequacy of the diet.^35^ Ruel stated that, lack of dietary diversity is a severe problem among populations whose diets are predominantly based on starchy staples and often include little or no animal products and few fresh fruits and vegetables.^36^ This current finding has shown that the vegetarians had low dietary diversity. A well-planned vegetarian diet that includes fortified foods can be nutritionally adequate for adults and children and can promote health and lower the risk of major chronic diseases. Larger studies on dietary intakes of vegetarians in Ghana will give a better understanding of their usual food intakes and assist in the formulation of evidencebased dietary guidelines for this population.

### Limitations

The use of self-reported dietary intakes may result in under-reporting or over-reporting of actual dietary intakes. The use of small sample size and participants from a particular religious denomination could also limit generalizability of the results.

## Conclusion

Participants had low dietary diversity. This may lead to inadequate nutrient intake in this group. There is the need for dietitians and nutritionists to provide appropriate education on well-planned vegetarian diets as well as emphasize on the importance of a variety for this type of diet.
